# An Improved System-Level Calibration Scheme for Rotational Inertial Navigation Systems

**DOI:** 10.3390/s22197610

**Published:** 2022-10-07

**Authors:** Qiushuo Wei, Feng Zha, Hongyang He, Bao Li

**Affiliations:** Department of Navigation, Naval University of Engineering, Wuhan 430033, China

**Keywords:** system-level calibration, systematic calibration, Kalman filter

## Abstract

The system-level calibration technology of rotational inertial navigation is one of the main methods to improve the accuracy of inertial navigation, and the design of the calibration scheme is the key to calibration technology. By the establishment of the error model of inertial navigation system, a 30-position calibration scheme is designed in this study. Based on the 30-dimensional Kalman filter, the constant errors, scale factor errors and installation error of gyroscope and accelerometer are identified. Comparing the traditional schemes and the 30-position scheme with the simulation experiment, the observability of the 30-position scheme is higher, the residual error of the estimated sensor is smaller and the navigation positioning accuracy after the estimated inertial sensor error parameter compensation is higher, which verifies the feasibility of the 30-position scheme. Finally, the measured experiment uses the 30-position scheme to estimate the error of a certain type of IMU sensor, and the calibration curve of the error parameter is well converged before the end of the calibration experiment, so it has certain practical value.

## 1. Introduction

The rotational inertial navigation system (RINS) is mainly composed of the inertial measurement unit (IMU), rotational mechanism and navigation computer. The measurement accuracy of IMU largely determines the navigation accuracy of the INS. Error calibration technology is one of the effective ways to compensate for the low accuracy of IMU [1,2,3,4,5,6,7]. Common error calibration techniques mainly include separate calibration and system-level calibration (also known as systematic calibration). Before the equipment is delivered, the experimenters will calibrate the IMU separately in the laboratory, and compensate the calibrated sensor errors, such as constant errors, scale factor errors, installation errors, etc. The compensated IMU sensor errors can be considered to have little impact on the navigation accuracy within a certain period of time. However, the IMU sensor errors will change with the passage of use time and the influence of the environment. In order to avoid excessive divergence of navigation errors, it is necessary to calibrate the IMU again. It is not appropriate to use the separate calibration method again, because the IMU needs to be disassembled, transported to the laboratory and re-equipped in the middle, which limits the possibility of long-distance voyages for the ship in terms of time and space. The system-level calibration technology can break the space–time limit of the split calibration, because it does not need the height reference of the turntable (the precision turntable provides position and velocity reference), and takes the navigation errors, such as attitude error, velocity error and position error, as the observation measurement, and combines this with the least square method or a filtering method, such as Kalman filter, to calibrate the error parameters [8,9,10,11,12,13,14,15,16,17,18].

The advantages and disadvantages of the system-level calibration scheme are mainly reflected in the observability of the error parameters. Therefore, designing a reasonable rotation scheme to stimulate the error parameters of the error related sensors is the key to the calibration. Xiaoxia Yang designed a group of carrier constraint motions, that is, the angular motion excitation error characteristics of rolling angle and yaw angle when the carrier moves in a uniform speed circle. The experimental results show that the motion constraint can calibrate 12 error parameters, including the constant errors and scale factor errors of gyroscope and accelerometer [19]. Wenfeng Shi designed a 10-position system-level calibration scheme, which can effectively estimate 24 inertial device error parameters [5]. Haina Weng designed a three-axis tilt calibration scheme, which can separate 30 relevant error parameters by comprehensively using the rotation and stop mode of gyroscope and accelerometer in the tilt state [15]. Zichao Wang et al. established a complex error parameter model of the strapdown inertial navigation system (SINS) for the error of the rod arm and the temperature error coefficient of the accelerometer, and proposed a system-level calibration method for complex error parameters [20]. The SAGEM company has designed an 18-position error calibration scheme. The calibration accuracy of this scheme meets the requirements of a navigation-level inertial navigation system, and is currently applied to the calibration of a laser gyro strapdown inertial navigation system [21].

Based on the previous calibration schemes, a 30-position rotation calibration scheme is designed in this paper. Based on the observation of velocity error and position error, the Kalman filter equation is established, which can estimate 21 error parameters, including constant errors, scale factor errors and installation errors of gyroscope and accelerometer. This paper is mainly developed from the following points: first, according to the principle of inertial navigation, the inertial sensor error equation and the systematic error equation are established; on this basis, a 30-dimensional Kalman filter is designed to achieve the full-parameter error calibration of the sensor error, and then a 30-position system-level calibration scheme is designed, and the observability analysis method of covariance is used to compare the excitation effect of the design scheme and the traditional scheme on the error parameters, and finally the proposed scheme is verified by simulation experiments and measured experiments.

## 2. IMU Error Model

### 2.1. Definition of Reference Frames

Inertial Frame (denoted as *i*): The origin of the inertial frame (denoted as *oi*) is located at the center of mass of the earth. The point from the origin to the north pole is defined as z-axis (denoted as *oi-zi*), the point from the origin to the average vernal equinox is x-axis (denoted as *oi-xi*), and the y-axis, z-axis and x-axis conform to the right-hand rule (denoted as *oi-yi*).

Earth Frame (denoted as *e*): The origin of the earth frame (denoted as *oe*) is located at the center of mass of the earth like the inertial frame. The point from the origin to the north pole is defined as the z-axis (denoted as *oe-ze*). The x-axis crosses the Greenwich meridian circle (denoted as *oe-xe*) through the origin in the equatorial plane. The y-axis, the z-axis and the x-axis conform to the right-hand rule (denoted as *oe-ye*).

Navigation Frame (denoted as *n*): The navigation frame is the local geographical coordinate system, and its origin (denoted as on) is located at the center of mass of the carrier. The direction specified by the x-axis is that the origin points to the East (denoted as *on-xn*), the direction specified by the y-axis is that the origin points to the North (denoted as *on-yn*) and the direction specified by the z-axis is that the origin points to the sky (denoted as *on-zn*).

Body Frame (denoted as *b*): The origin of the body frame (denoted as *ob*) is also located at the center of mass of the carrier. The direction designated by the x-axis is that the origin points to the right side of the carrier (marked as *ob-xb*), the direction designated by the y-axis is that the origin points to the front side of the carrier (denoted as *ob-yb)*, and the direction designated by the z-axis is that the origin points to the top of the carrier (denoted as *ob-zb*).

IMU Frame (denoted as *p*): In the SINS, the frame *p* coincides with the frame *b*, so it can be considered that the two frames are the same frame, but in the RINS, the IMU rotation causes the frame *p* to have relative motion with the frame *b*, so the frame *p* needs to be defined. The IMU is orthogonally mounted by three sets of gyroscopes and accelerometers, with its origin as *op*, and the three sets of inertial sensors pointing to *op-xp*, *op-yp* and *op-zp* respectively.

### 2.2. Definition of System Errors

Constant Errors (denoted as ε and ∇): The physical meaning of constant errors are the value output when IMU has no input. The constant error of gyroscope and accelerometer can be expressed as follows:

(1) $$\varepsilon ={\left[\begin{array}{ccc} \varepsilon_{x} & \varepsilon_{y} & \varepsilon_{z} \end{array}\right]}^{T}$$

(2) $$\nabla ={\left[\begin{array}{ccc} \nabla_{x} & \nabla_{y} & \nabla_{z} \end{array}\right]}^{T}$$

Scale Factor Errors (denoted as $\delta K_{g}$ and $\delta K_{a}$): The physical meaning of scale factor errors are the ratio error between the output value and the input value of IMU, in ppm.

(3) $$\delta K_{g}=\left[\begin{array}{ccc} \delta K_{gx} & 0 & 0\\ 0 & \delta K_{gy} & 0\\ 0 & 0 & \delta K_{gz} \end{array}\right]$$

(4) $$\delta K_{a}=\left[\begin{array}{ccc} \delta K_{ax} & 0 & 0\\ 0 & \delta K_{ay} & 0\\ 0 & 0 & \delta K_{az} \end{array}\right]$$

Installation Errors (denoted as $\delta A_{g}$ and $\delta A_{a}$): IMU is composed of three groups of gyroscopes and accelerometers. Theoretically, the three groups of inertial sensors should be installed orthogonally. Due to the problem of processing accuracy, it is impossible to install IMU orthogonally. There will be a small angle between the actual installation coordinate system and the ideal IMU frame, which is the installation errors, and the unit is ″. Under normal circumstances, the installation error angles of the accelerometer and the gyroscope are all 6. In order to reduce the number of calibration parameters, reduce the complexity of the algorithm and improve the calibration rate, it is often assumed that the ox axis of the orthogonal ideal IMU frame coincides with the input axis ox of the gyroscope, the oy axis is in the plane oxy determined by the input axis ox of the x gyroscope and the input axis oy of the y gyroscope, and the oz axis can be determined by the right-hand rule. The installation error matrix is as follows:

(5) $$\delta A_{g}=\left[\begin{array}{ccc} 0 & 0 & 0\\ \delta A_{gyx} & 0 & 0\\ \delta A_{gzx} & \delta A_{gzy} & 0 \end{array}\right]$$

(6) $$\delta A_{a}=\left[\begin{array}{ccc} 0 & \delta A_{axy} & \delta A_{axz}\\ \delta A_{ayx} & 0 & \delta A_{ayz}\\ \delta A_{azx} & \delta A_{azy} & 0 \end{array}\right]$$

### 2.3. Establishment of IMU Error Model

Ignoring the random errors, let $\omega_{ib}^{b}$ be the true angular rate output of the gyroscope without error under frame *b*, and $f_{ib}^{b}$ be the true specific force output of the accelerometer without error under frame *b*. The IMU error equation caused by the error source in Section 2.2 is as follows:

(7) $$\left[\begin{array}{c} \delta \omega_{ibx}^{b}\\ \delta \omega_{iby}^{b}\\ \delta \omega_{ibz}^{b} \end{array}\right]=\left[\begin{array}{c} \varepsilon_{x}\\ \varepsilon_{y}\\ \varepsilon_{z} \end{array}\right]+\left[\begin{array}{ccc} \delta K_{gx} & 0 & 0\\ \delta A_{gyx} & \delta K_{gy} & 0\\ \delta A_{gzx} & \delta A_{gzy} & \delta K_{gz} \end{array}\right]\left[\begin{array}{c} \omega_{ibx}^{b}\\ \omega_{iby}^{b}\\ \omega_{ibz}^{b} \end{array}\right]$$

(8) $$\left[\begin{array}{c} \delta f_{ibx}^{b}\\ \delta f_{iby}^{b}\\ \delta f_{ibz}^{b} \end{array}\right]=\left[\begin{array}{c} \nabla_{x}\\ \nabla_{y}\\ \nabla_{z} \end{array}\right]+\left[\begin{array}{ccc} \delta K_{ax} & \delta A_{axy} & \delta A_{axz}\\ \delta A_{ayx} & \delta K_{ay} & \delta A_{ayz}\\ \delta A_{azx} & \delta A_{azy} & \delta K_{az} \end{array}\right]\left[\begin{array}{c} f_{ibx}^{b}\\ f_{iby}^{b}\\ f_{ibz}^{b} \end{array}\right]$$

## 3. System-Level Calibration Based on 30-Dimensional Kalman Filter

### 3.1. Establishment of Kalman Filter

In order to calibrate all the error parameters in the IMU error model in the previous section, an online calibration method based on a 30-dimensional Kalman filter was designed in this paper. The Kalman filter recursively estimated the error parameters of IMU by observing the velocity error and position error of the navigation system. The Kalman filter was established by the following error propagation equation of strapdown inertial navigation system.

(9) $$\dot{\phi }=-\omega_{in}^{n}\times \phi +\delta \omega_{in}^{n}-C_{b}^{n}\delta \omega_{ib}^{b}$$

(10) $$\begin{array}{cc} \delta {\dot{v}}^{n} & =C_{b}^{n}f^{b}\times \phi^{n}+C_{b}^{n}\delta f^{b}-\left(2\omega_{ie}^{n}+\omega_{en}^{n}\right)\times \delta v^{n}\\ & -\left(2\delta \omega_{ie}^{n}+\delta \omega_{en}^{n}\right)\times v^{n}-\delta g \end{array}$$

(11) $$\left\{\begin{array}{l} \delta \dot{L}=\frac{\delta v_{N}}{R_{M}+h}-\frac{v_{N}\cdot \delta h}{{\left(R_{M}+h\right)}^{2}}\\ \delta \dot{\lambda }=\frac{\sec L\cdot \delta v_{E}}{R_{N}+h}+\frac{v_{E}\cdot \tan L\sec L\cdot \delta L}{R_{N}+h}-\frac{v_{E}\sec L\cdot \delta h}{{\left(R_{N}+h\right)}^{2}}\\ \delta \dot{h}=\delta v_{U} \end{array}\right.$$

where $\phi ={\left[\begin{array}{ccc} \phi_{E}^{n} & \phi_{N}^{n} & \phi_{U}^{n} \end{array}\right]}^{T}$ represents the attitude of the INS in frame *n*; $v^{n}={\left[\begin{array}{ccc} v_{E}^{n} & v_{N}^{n} & v_{U}^{n} \end{array}\right]}^{T}$ represents the speed of the INS in frame *n*; L,λ,h represents the position of the INS in the frame *n*; $\omega_{ie}^{n}$ is the earth rotation angle velocity in the frame *n*; $\omega_{en}^{n}$ is the projection of the rotational angular velocity of the frame *n* relative to the frame *e* under the frame *n*, and $C_{b}^{n}$ is the rotation matrix from the frame *b* to the frame *n*; $R_{M}$ is the radius of curvature of the meridian circle, and $R_{N}$ is the radius of curvature of the prime unitary circle; δg is the local gravitational acceleration error.

The INS error propagation equation was sorted into matrix form, and the state equation of the Kalman filter was obtained by arranging the matrix as follows:

(12) $$\dot{X}\left(t\right)=F\left(t\right)X\left(t\right)+G\left(t\right)W\left(t\right)$$

where $X\left(t\right)$ is a 30-dimensional state vector, which is specifically expressed as follows:

(13) $$\begin{array}{cc} X\left(t\right)= & [\varphi_{E}\;\varphi_{N}\;\varphi_{U}\;\delta v_{E}\;\delta v_{N}\;\delta v_{U}\;\delta L\;\delta \lambda \;\delta h\;\varepsilon_{x}\;\varepsilon_{y}\;\varepsilon_{z}\\ & \nabla_{x}\;\nabla_{y}\;\nabla_{z}\;\delta K_{gx}\;\delta K_{gy}\;\delta K_{gz}\;\delta K_{ax}\;\delta K_{ay}\;\delta K_{az}\\ & \delta A_{gyx}\;\delta A_{gzx}\;\delta A_{gzy}\;\delta A_{ayx}\;\delta A_{azx}\;\delta A_{axy}\;\delta A_{azy}\;\delta A_{axz}\;\delta A_{ayz}{]}^{T} \end{array}$$

$\dot{X}\left(t\right)$ is the differential matrix of the state vector; $F\left(t\right)$ is the INS transfer matrix, which can be determined by the expanded INS error propagation equation; $G\left(t\right)$ is the noise distribution matrix of INS; $W\left(t\right)$ is the noise matrix of INS.

The observation equation of Kalman filter was as follows. In this paper, velocity error and position error were selected as the observation:

(14) $$Z\left(t\right)=H\left(t\right)X\left(t\right)+V\left(t\right)$$

where $Z\left(t\right)$ is the observation measurement composed of system velocity error and position error, $H\left(t\right)$ is the system observation matrix, and $V\left(t\right)$ is the velocity position observation noise matrix. The specific expansion of the above matrix was as follows:

(15) $$Z\left(t\right)={\left[\begin{array}{llllll} \delta v_{E} & \delta v_{N} & \delta v_{U} & \delta L & \delta \lambda & \delta h \end{array}\right]}^{T}$$

(16) $$H\left(t\right)={\left[\begin{array}{llll} 0_{3\times 3} & I_{3\times 3} & 0_{3\times 3} & 0_{3\times 21}\\ 0_{3\times 3} & 0_{3\times 3} & I_{3\times 3} & 0_{3\times 21} \end{array}\right]}_{6\times 30}$$

### 3.2. Calibration Arrangement Scheme

The design of the rotation calibration scheme is the key to calibration. Different rotation path arrangement has different excitation effects on the sensor errors of IMU. Therefore, a reasonable rotation calibration scheme must meet two basic requirements: one is that it should be able stimulate the more complete sensor error parameters of IMU, and the other is that the observability of error parameters should be high. Therefore, according to IMU error model, error propagation characteristics of INS and observability analysis, this paper designed a 30-position rotation calibration scheme as shown in Table 1. Only a part of the rotation scheme is shown in the table, and the other part of the rotation scheme has the same path as the previous part, and the rotation direction is opposite. This scheme can calibrate 21 related sensor errors, including constant error, scale factor error and installation error of gyroscopes and accelerometers.

### 3.3. INS Observability Analysis

Two common observability analysis methods are observable singular value decomposition analysis (SVD) and observable covariance analysis. The observable covariance method uses the diagonal change of the diagonal elements of the mean variance matrix of the Kalman filter equation over time to observe the estimation of the corresponding state components. In the next section of the simulation verification experiment, the observability was solved by the covariance method, the principle of which is shown below.

Discretization of the established Kalman filter model was as follows:

(17) $$\left\{\begin{array}{l} X_{k}=F_{k/k-1}X_{k-1}+\Gamma_{k/k-1}W_{k-1}\\ Z_{k}=H_{k}X_{k}+V_{k} \end{array}\right.$$

The prediction and recurrence process of Kalman filter was as follows:

State one-step prediction:

(18) $${\hat{X}}_{k/k-1}=F_{k/k-1}{\hat{X}}_{k-1}$$

State one-step prediction mean square error matrix:

(19) $$P_{k/k-1}=F_{k/k-1}P_{k-1}F_{k/k-1}^{T}+\Gamma_{k-1}Q_{k-1}\Gamma_{k-1}^{T}$$

Filter gain calculation:

(20) $$K_{k}=P_{k/k-1}H_{k}^{T}{\left(H_{k}P_{k/k-1}H_{k}^{T}+R_{k}\right)}^{-1}$$

State estimation:

(21) $${\hat{X}}_{k}={\hat{X}}_{k/k-1}+K_{k}\left(Z_{k}-H_{k}{\hat{X}}_{k/k-1}\right)$$

Mean square error of state estimation:

(22) $$P_{k}=\left(I-K_{k}H_{k}\right)P_{k/k-1}$$

where $Q_{k}$ represents the mean square error matrix of the output white noise of the gyroscope and accelerometer, and $R_{k}$ represents the variance matrix of the observation noise. In general, it is required that $Q_{k}$ is non-negative definite and $R_{k}$ is positive definite.

The observability calculation method based on covariance analysis was defined as follows [22]:

(23) $$\sigma_{k\left(j\right)}=\sqrt{\frac{P_{0\left(jj\right)}}{P_{k\left(jj\right)}}}$$

where $P_{0\left(jj\right)}$ represents the value corresponding to a certain state component in the diagonal element of the initial mean square error matrix $P_{0}$ of the Kalman filter, and the initial mean square error matrix is usually set according to the empirical value. $P_{k\left(jj\right)}$ represents the value corresponding to a certain state component in the diagonal element of the Kalman filter mean square error matrix $P_{k}$ at a certain time. With the progress of filtering, the error of the state quantity becomes smaller and smaller, and $P_{k}$ also becomes smaller. Therefore, the value of $\sigma_{k\left(j\right)}$ becomes larger as the estimation error of the state quantity decreases.

According to experience, the observability of the state component can be roughly determined by setting the following thresholds:

(24) $$\left\{\begin{array}{cc} \sigma_{k\left(j\right)}\leq 1 & \mathrm{unobservable}\\ 1<\sigma_{k\left(j\right)}\leq 2 & \mathrm{low}-\mathrm{observability}\\ 2<\sigma_{k\left(j\right)}\leq 10 & \mathrm{moderate}-\mathrm{observability}\\ \sigma_{k\left(j\right)}>10 & \mathrm{high}-\mathrm{observability} \end{array}\right.$$

## 4. Verification of Simulations and Experiments

### 4.1. Simulations Verification

SAGEM has designed an 18-position calibration scheme that is a common method for calibrating laser gyro-jet inertial navigation systems, which has been tested by engineering practice and can achieve the calibration accuracy requirements of navigation-level inertial systems in a short period of time [21].

In order to verify the calibration effect of the 30-position scheme of the design, a simulation experiment was set up to verify the feasibility. The initialization parameters of the simulation experiment were set as follows: longitude and latitude (114°, 30°), initial attitude of the 18-position scheme (0, −180°, −90°), initial attitude of the 30-position scheme (0, −90°, −90°) and the initial speed 0.

The quantitative comparison of simulation observability between the 18-position scheme and the 30-position calibration scheme designed in this paper was as follows:

It can be seen from formula (24) and Table 2 that the observability of the two schemes was at the level of “strong”, and the observation degree corresponding to the state component of the 30-position scheme was mostly stronger than that of the 18-position scheme. Therefore, the 30-position scenario estimate from the observability point of view was more accurate.

**Figure 1:**
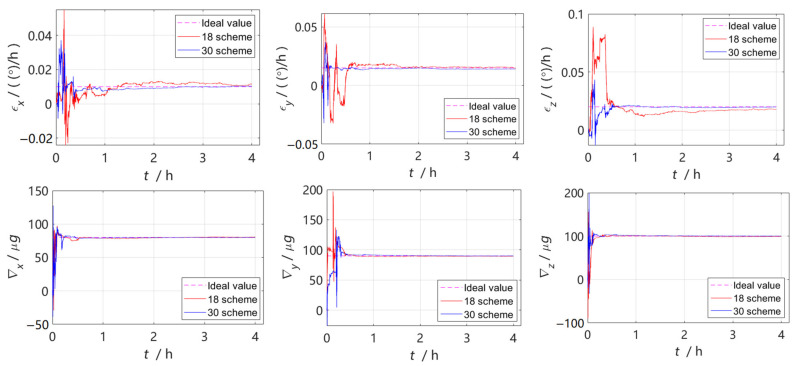

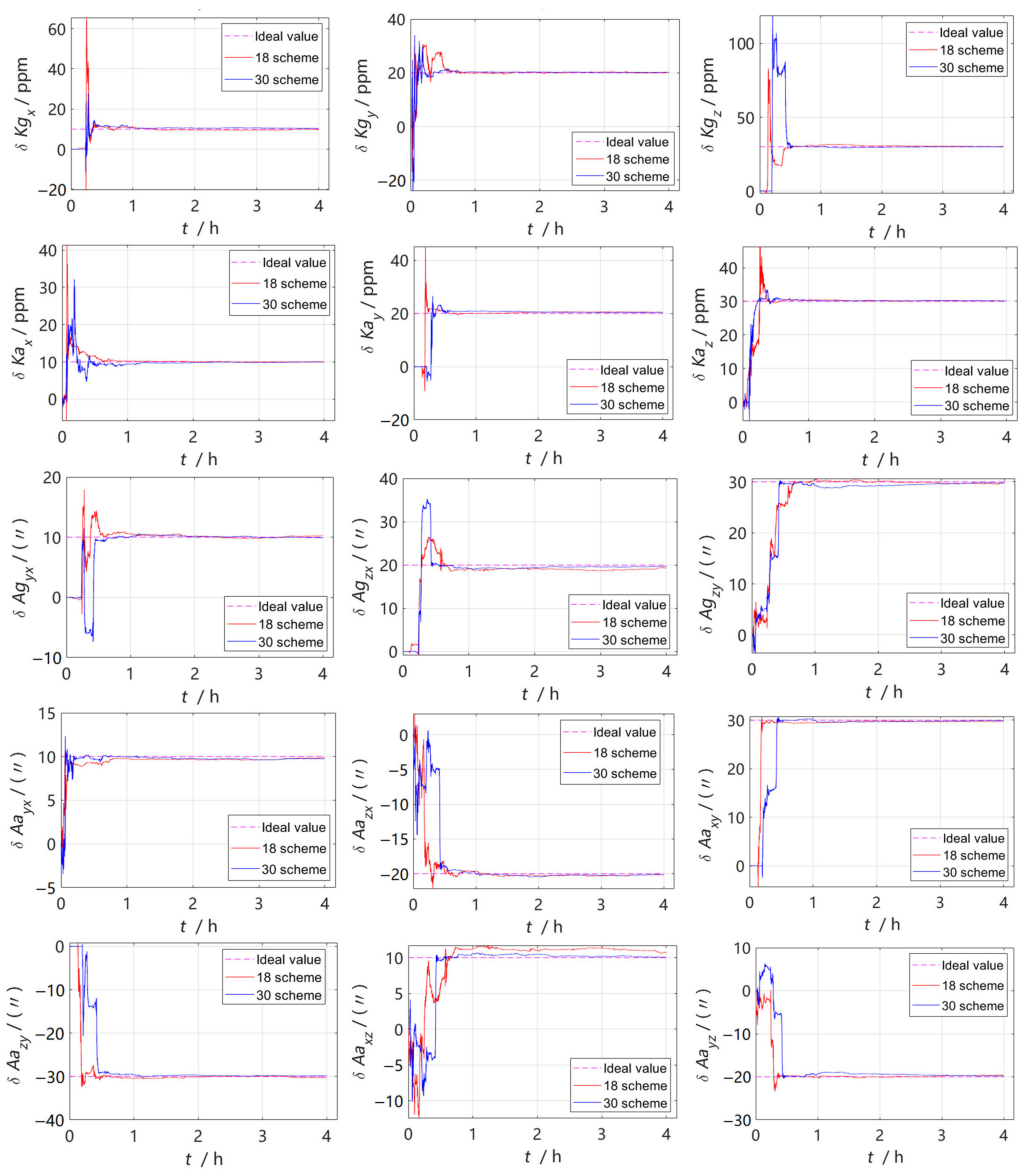
Error parameter estimation curve in simulation.

The Kalman filter estimation curve is shown in Figure 1, where the pink dotted line represents the ideal error value set, the red solid line represents the calibration curve of the 18-position scheme, and the blue solid line represents the calibration curve of the 30-position scheme. From the convergence effect, most of the error parameters of the 30-position scheme converged quickly, of which the gyroscope constant error and part of the installation error convergence effect were the most obvious.

**Figure 2:**
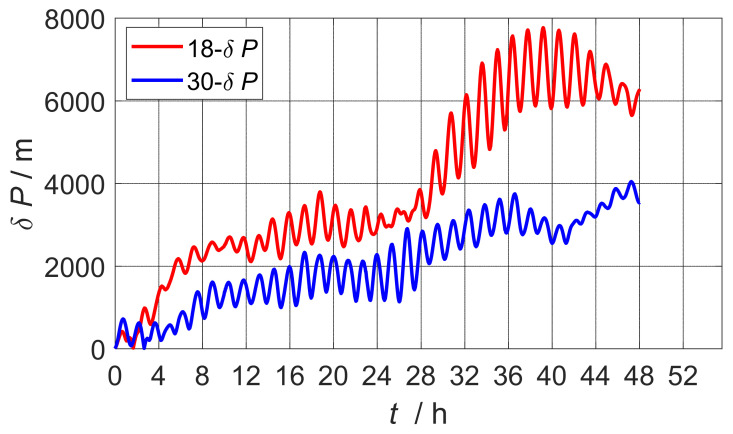
Comparison of positioning error of navigation after calibration compensation of 30-position scheme and 18-position scheme.

The estimation results of the error parameters of the calibration simulation experiment are shown in Table 3. The table lists the error calibration results and ideal values for the two calibration schemes, as well as the residual errors calculated on their basis. Comparing the simulation estimates, it can be seen that the residual error of the 30-position scheme was smaller than the residual error of the 18-position scheme, so the scheme proposed in this paper had stronger calibration accuracy. Figure 2 shows the comparison curve of the static base pure inertial navigation positioning error after 48 h of inertia sensor error compensation estimated by the two calibration schemes. The maximum position error of the 30-position scheme designed in the paper was reduced by 3639 m, which verified that the proposed scheme was better.

**Figure 3:**
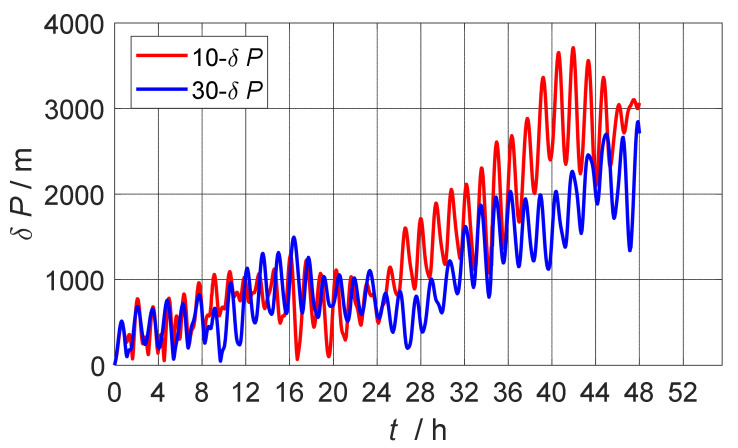
Comparison of positioning error of navigation after calibration compensation of 30-position scheme and 10-position scheme.

In order to more fully illustrate that the 30-position calibration scheme designed herein has a good calibration effect, this paper also carried out the experimental comparison of the 30-position scheme and the 10-position scheme [5], and the experimental results are shown in Figure 3. After calibration compensation, 48-h static base pure inertial navigation was carried out, and the positioning error of the 30-position scheme was 865 m smaller than that of the 10-position scheme. Therefore, it was fully illustrated that the 30-position scheme was better.

### 4.2. Experiments Verification

**Figure 4:**
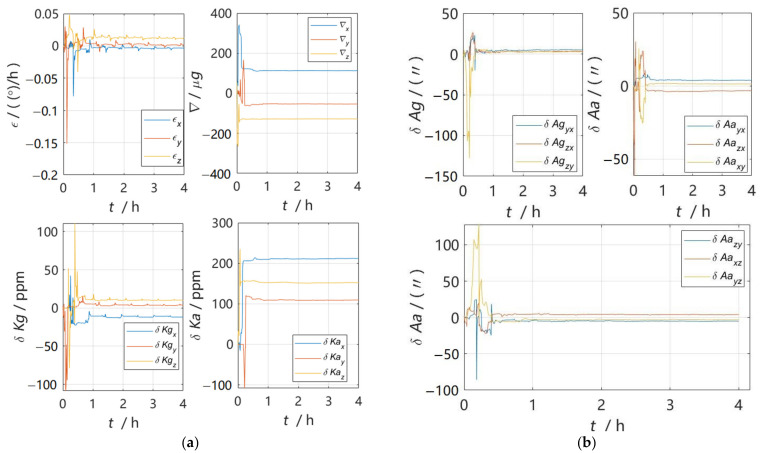
Error parameter estimation curve. (**a**) Constant error and scale factor error estimation curve of gyroscope and accelerometer; (**b**) installation error estimation curve of gyroscope and accelerometer.

The above simulation experiment shows that the 30-position scheme designed in this paper is better than the traditional scheme from three aspects: observability analysis, residual error comparison and navigation positioning error comparison after inertial sensor error parameter compensation. In order to test the practical application value of the 30-position scheme, the physical experimental data collection was carried out using a certain model IMU. The error estimation curve after data processing is shown in Figure 4, and it can be seen from the figure that the error calibration curve achieved good convergence and verified the practical value of the 30-position calibration scheme. The IMU error parameter estimation results were as shown in Table 4.

## 5. Conclusions

In this paper, a 30-position system-level calibration scheme was designed based on the error characteristics of IMU and the error propagation equation of inertial navigation system. In order to illustrate the good calibration effect of the designed calibration arrangement, this paper analyzed the observability, residual error and navigation positioning error after compensation of the inertial sensor error parameters of the proposed and traditional schemes through simulation experiments, and the simulation experiment results showed that the observability of the 30-position scheme was stronger, the residual error was smaller and the estimated navigation error after inertial sensor error compensation was smaller, which verified the feasibility of the 30-position scheme. In addition, in order to verify the engineering practical value of the 30-position scheme, the rotary table and IMU were used in the laboratory to conduct actual measurement experiments, and from the data processing results, the error parameter estimation curve was found to converge well and to have certain engineering practical value.

## Figures and Tables

**Table 1:** 30-position rotation scheme.

Rotation Order	Rotation Scheme		Rotation Order	Rotation Scheme	
	Frame *n*	Frame IMU		Frame *n*	Frame IMU
1	E + 90°	y + 90°	9	E + 90°	x + 90°
2	E + 90°	y + 90°	10	E − 180°	x − 180°
3	E + 90°	y + 90°	11	E − 90°	x − 90°
4	E − 180°	y − 180°	12	N + 90°	z + 90°
5	E − 90°	y − 90°	13	N + 90°	z + 90°
6	N + 90°	z + 90°	14	N − 180°	z − 180°
7	E + 90°	x + 90°	15	N − 90°	z − 90°
8	E + 90°	x + 90°	The order (16~30) is the same as that of (1~15), and the direction is opposite		

**Table 2:** Comparison of observability between two schemes.

Parameter	18-Position Scheme	30-Position Scheme
$\varepsilon_{x}$	22.37	79.94
$\varepsilon_{y}$	23.08	69.69
$\varepsilon_{z}$	25.05	75.55
$\nabla_{x}$	181.52	251.99
$\nabla_{y}$	197.31	263.65
$\nabla_{z}$	170.47	247.22
$\delta K_{gx}$	31.99	80.49
$\delta K_{gy}$	65.02	95.85
$\delta K_{gz}$	103.45	186.40
$\delta K_{ax}$	64.07	154.33
$\delta K_{ay}$	163.65	318.26
$\delta K_{az}$	103.46	225.95
$\delta A_{gyx}$	111.99	100.50
$\delta A_{gzx}$	158.46	155.51
$\delta A_{gzy}$	165.26	282.83
$\delta A_{ayx}$	274.16	332.01
$\delta A_{azx}$	229.89	261.05
$\delta A_{axy}$	452.96	633.62
$\delta A_{azy}$	54.74	102.71
$\delta A_{axz}$	174.74	244.61
$\delta A_{ayz}$	22.37	79.94

**Table 3:** The error parameter estimation results in simulation.

Error Parameters	Ideal Value	18-Estimate Value	30-Estimate Value	18-Residual Value	30-Residual Value
ε°h	0.01	0.011301017	0.010314397	0.001301017	0.000314397
	0.015	0.014783523	0.014088555	−0.000216477	−0.000911445
	0.02	0.017859505	0.019863169	−0.002140495	−0.000136831
$\nabla \;\left(\mathrm{ug}\right)$	80	80.45810632	79.83330178	0.458106325	−0.166698218
	90	89.58384702	89.98982828	−0.416152981	−0.010171722
	100	99.2916787	100.3147819	−0.708321297	0.314781869
$\delta K_{g}\left(\mathrm{ppm}\right)$	10	9.787512187	10.40013812	−0.212487813	0.400138118
	20	20.15702346	20.20475451	0.157023456	0.20475451
	30	30.18298086	29.97072048	0.182980864	−0.029279523
$\delta K_{a}\left(\mathrm{ppm}\right)$	10	10.07074401	9.96198043	0.070744014	−0.03801957
	20	20.44490916	20.4155934	0.444909159	0.415593405
	30	30.15649279	30.15181757	0.156492792	0.151817574
$\delta {A_{g}}_{\left(\prime \prime \right)}$	10	10.28440017	9.906793199	0.284400167	−0.093206801
	20	19.36711027	19.67128246	−0.632889727	−0.328717541
	30	29.76259989	29.78869477	−0.237400115	−0.211305227
$\delta {A_{a}}_{\left(\prime \prime \right)}$	10	9.902931167	9.750802654	−0.097068833	−0.249197346
	−20	−20.1225843	−20.12456347	−0.122584303	−0.12456347
	−30	−30.28877323	−29.80122684	−0.288773226	0.198773161
	30	29.75675589	29.92631969	−0.243244114	−0.073680314
	10	10.67119009	10.06981142	0.67119009	0.069811417
	−20	−19.61482211	−19.78829016	0.385177888	0.211709844

**Table 4:** The error parameter estimate of the IMU.

Error Parameter	Calibration Value		
Constant error of gyroscope (°/h)	−0.004108297	0.001381599	0.012232855
Constant error of accelerometer (ug)	112.5559752	−53.77119242	−128.2295866
Scale factor error of gyroscope (ppm)	−11.72975729	3.722942935	10.07015303
Scale factor error of accelerometer (ppm)	211.7416169	109.248313	152.0861156
Installation error of gyroscope (″)	5.148117571	3.244895406	2.732270054
Installation error of accelerometer (″)	3.801944879	−3.359659629	−5.07799273
	1.355994872	3.9076545111	−2.908936285

## Data Availability

This study did not report any data.
